# Plasmapheresis as a Bridge to Urgent Thyroidectomy in Severe Amiodarone-Induced Thyrotoxicosis

**DOI:** 10.7759/cureus.92355

**Published:** 2025-09-15

**Authors:** Roneil N Parikh, Emma Verner, Avinash Suryawanshi, Kirtan Ganda, Erick Fuentes

**Affiliations:** 1 Breast and Endocrine Surgery, Concord Repatriation General Hospital, Sydney, AUS; 2 Concord Clinical School, The University of Sydney, Sydney, AUS; 3 Hematology, Concord Repatriation General Hospital, Sydney, AUS; 4 Endocrinology and Metabolism, Concord Repatriation General Hospital, Sydney, AUS; 5 Breast and Endocrine surgery, Concord Repatriation General Hospital, Sydney, AUS

**Keywords:** amiodarone induced thyrotoxicosis, case report, plasma exchange, plasmapheresis, thyroidectomy

## Abstract

Amiodarone-induced thyrotoxicosis (AIT) is a challenging condition to manage due to the drug's long half-life and frequent resistance to conventional medical therapy. Plasmapheresis has emerged as a therapeutic option in patients requiring urgent thyroidectomy.

We present the case of a 68-year-old male with a history of atrial fibrillation treated with amiodarone for two years, who presented with diarrhea, weight loss, and fever. Clinical examination revealed tachycardia and pyrexia, without goitre or ophthalmopathy. Laboratory tests showed thyrotoxicosis (free T4 >100 pmol/L), deranged liver function tests, and negative thyroid-stimulating hormone (TSH)-receptor antibodies. Imaging with abdominal CT revealed a 10.7 cm lesion in the left hepatic lobe, suspicious for ruptured hepatocellular carcinoma (HCC), warranting a planned left hemi-hepatectomy. Thyroid ultrasound demonstrated mild glandular enlargement without nodules, consistent with type 2 AIT.

Despite medical therapy, the patient's thyrotoxic symptoms worsened, with persistent fever, tachycardia, and tremors. He underwent three sessions of plasmapheresis, which served as a bridge to total thyroidectomy, to reduce the risk of intraoperative thyroid storm. The postoperative course was uncomplicated, and histology confirmed type 2 AIT. Two weeks later, the patient underwent successful left hemi-hepatectomy, with pathology confirming moderately differentiated HCC.

AIT can exacerbate pre-existing cardiac conditions and is associated with increased morbidity and mortality. While initial management typically involves conventional therapy, patients with severe disease or those requiring urgent surgical interventions may benefit from timely plasmapheresis as a bridging strategy to definitive thyroidectomy. The optimal timing, number of exchanges, and choice of replacement fluid remain areas for further research.

## Introduction

Amiodarone is an iodine-rich drug that can cause changes in thyroid function tests due to its pharmacological properties. Amiodarone-induced thyrotoxicosis (AIT) is a disorder characterised by a hyperthyroid state resulting from either uncontrolled autonomous thyroid hormone production (type I) or destructive thyroiditis (type II). Severe thyrotoxicosis and thyroid storm are life-threatening conditions, particularly in patients with underlying cardiac disease [1]. Type II AIT is an uncommon cause of thyroid storm and is often difficult to manage due to the prolonged half-life of amiodarone and resistance to conventional therapies, including antithyroid medications and corticosteroids [2].

Emergency total thyroidectomy is recommended for AIT patients with worsening cardiac function unresponsive to medical management [1, 3-4]. Rapidly restoring a euthyroid state improves cardiac function in patients with severe left ventricular systolic dysfunction, thereby reducing the risk of mortality [1]. Plasmapheresis or plasma exchange is a therapeutic intervention that involves extracorporeal removal, return, or exchange of blood plasma or components.

We present a rare case of type II AIT requiring plasmapheresis as a bridging therapy to emergency thyroidectomy due to refractory thyrotoxicosis, concern for intraoperative thyroid storm, and a concurrent ruptured hepatocellular carcinoma necessitating urgent hepatectomy. We also discuss the role and current evidence for plasmapheresis in managing severe AIT and address the optimal timing of surgery in complex cases such as this. This case has been reported in accordance with the CARE guidelines [5].

## Case presentation

A 68-year-old man initially presented with a two-day history of diarrhea and fever. His medical history included atrial fibrillation, hypertension, and dyslipidemia. Stool culture was positive for *Campylobacter*, and he was treated with a three-day course of azithromycin. Liver function tests were deranged with bilirubin of 22 mg/dl, GGT 57 U/L, ALP 78 U/L, ALT 623U/L, and AST 234U/L. Liver ultrasound revealed two heterogeneous hyperechoic lesions - one (L1) occupying the left hepatic lobe measuring 10.7 × 10.7 × 9.8 cm, and a second (L2) in segments 7/8 measuring 5.2 × 4.8 × 4.2 cm.

**Figure 1:**
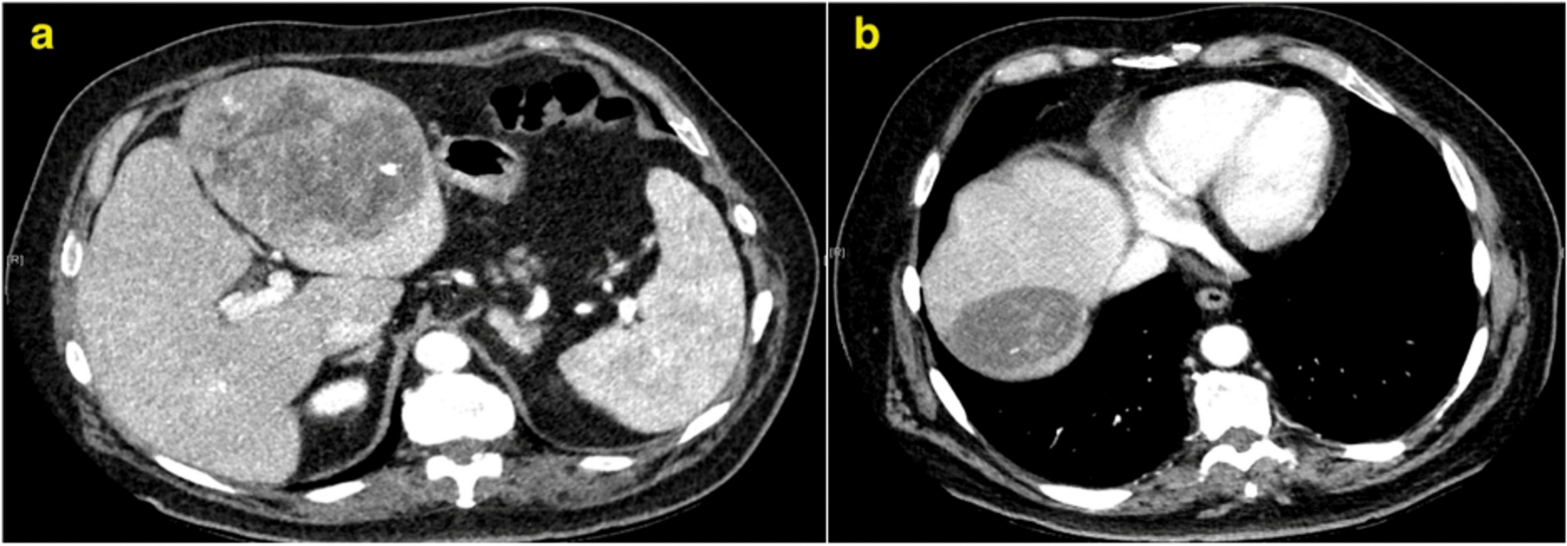
Triple phase computed tomography (CT) axial view of the liver demonstrating a well-defined heterogenous mass in the left lobe with intense arterial enhancement and no washout with areas of haemorrhage suspicious for a ruptured hepatocellular carcinoma (HCC) (Figure 1a). Lesion in right lobe of liver, more well-defined, heterogenous with focal calcification, later identified as a hydatid cyst (Figure 1b).

Triple-phase computed tomography (CT) of the liver showed L1 as a well-defined, heterogeneous mass in the left lobe with intense arterial enhancement and no washout. Importantly, areas of haemorrhage posterior to this lesion raised concern for a ruptured hepatocellular carcinoma (HCC) (Figure 1a). L2 was also well-defined but exhibited focal calcification and a different imaging profile, suggesting a separate pathology (Figure 1b). The patient was discharged with a plan for elective left hemi-hepatectomy

Two weeks later, he was re-admitted with persistent diarrhoea, profound weight loss (~20kg), diaphoresis, presyncope, and rapid atrial fibrillation. He denied heat intolerance. There were no symptoms to suggest ophthalmopathy or a history of recent iodinated contrast. There was no neck pain. The patient had been on amiodarone 200 mg daily for three years for atrial fibrillation, and was recently increased to 200 mg three times daily.

On examination, his heart rate was 90 bpm, and he had a postural drop in blood pressure from 130/80 mm Hg standing to 97/70 mm Hg supine. His oxygen saturation (98%) and temperature (36.7 C) were within normal limits. His thyroid gland was not enlarged, and there was no neck tenderness. There were no bruits or signs of ophthalmopathy. There were no signs of cardiac failure. Abdominal examination revealed hepatomegaly with the liver border 3cm below the right costal margin.

**Figure 2:**
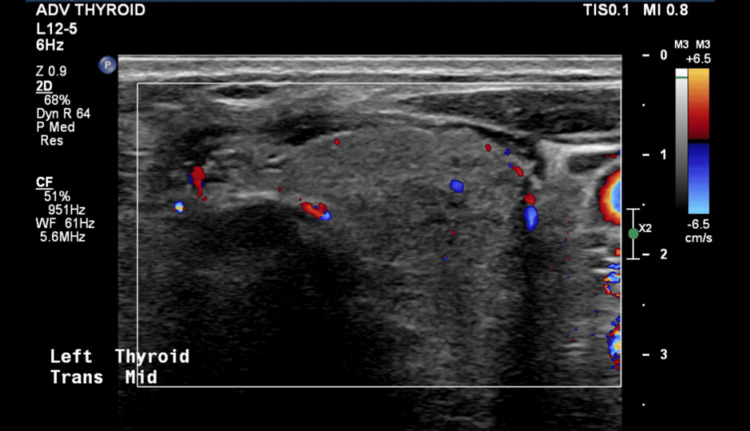
Ultrasound of the left thyroid lobe demonstrating mildly enlarged heterogeneous gland with mildly reduced vascularity

Thyroid function tests revealed thyrotoxicosis with suppressed thyroid-stimulating hormone (TSH) <0.02mIU/L, and significantly elevated free T4 >100pmol/L and free T3 19.2pmol/L with negative thyroid antibodies (TRAB). Thyroid ultrasound demonstrated a mildly enlarged, heterogeneous gland with reduced vascularity (Figure 2).

Amiodarone was discontinued in consultation with the cardiology team, and treatment with carbimazole 20 mg TDS and metoprolol 50 mg BD was initiated. Liver biopsy confirmed L1 as a probable HCC and L2 as a hydatid cyst; albendazole was started for the latter.

Despite medical therapy, the patient's thyrotoxicosis worsened, with persistent fever, tachycardia, and tremors. Carbimazole was increased to 20 mg QID, and prednisone 50 mg daily was added, with minimal clinical improvement. In light of the urgent need for HCC resection and concerns about wound healing with corticosteroids, both prednisone and apixaban were ceased.

A multidisciplinary team involving endocrinology, haematology, cardiology, anaesthesia, and hepatobiliary surgery recommended bridging plasmapheresis to reduce the risk of intraoperative thyroid storm. A total thyroidectomy was planned prior to hepatectomy. Metoprolol was switched to propranolol to reduce peripheral T4 to T3 conversion and modulate systemic vascular resistance.

**Figure 3:**
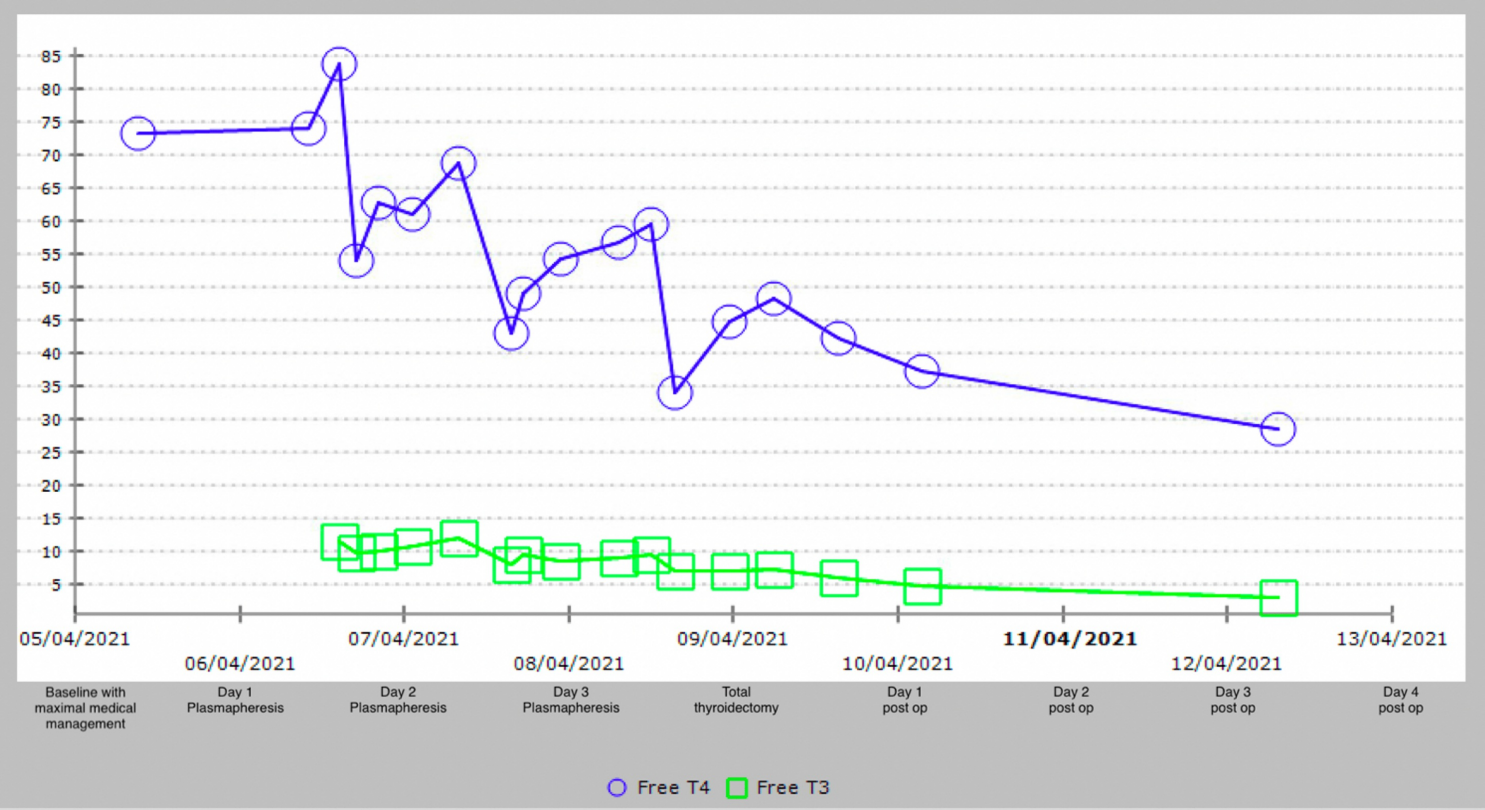
Trend of free thyroxine in pmol/L (FT4) and free triiodothyronine in pmol/L (FT3) through diagnosis, plasmapheresis and surgery. It demonstrates an increase in the FT4 levels between cycles of plasmapheresis, with an overall reduction highlighting the importance of timing the surgical management after completion of plasmapheresis.

The patient underwent three consecutive days of plasmapheresis, followed by 100 mg hydrocortisone TDS post-procedure. Thyroid function was monitored every six hours, showing both immediate and sustained reductions in free T3 and T4 over three days, with improvement in his clinical condition (Figure 3). With a reduction in his thyroid hormone levels as a result of each plasmapheresis cycle, there was demonstrated clinical improvement with a resolution of his tremors, fevers, and tachycardia. Albumin was used in the earlier sessions as the replacement fluid, followed by fresh frozen plasma (FFP) during the final session to prevent coagulopathy in the setting of urgent surgery. He underwent an uncomplicated total thyroidectomy, followed by HDU monitoring. Postoperatively, thyroid hormone levels normalized, and he was discharged after 10 days. Three weeks later, he underwent a successful open left hemi-hepatectomy. Histology confirmed the lesion to be a moderately differentiated HCC.

## Discussion

Amiodarone is commonly prescribed in the management of supraventricular arrhythmias. Due to its high iodine content and complex pharmacokinetics, it can significantly affect thyroid function, leading to amiodarone-induced hypothyroidism (AIH) or amiodarone-induced thyrotoxicosis (AIT) in up to 15% of patients [1, 6]. Discontinuation of amiodarone may be considered in patients with non-life-threatening arrhythmias; however, due to its long half-life and extensive tissue distribution, cessation alone has a limited impact on short-term thyroid outcomes [7, 8].

Type I AIT, often occurring in patients with underlying thyroid pathology such as nodular goitre or latent Graves' disease, is a form of iodine-induced hyperthyroidism. It is characterized by an increased, autonomous biosynthesis of thyroid hormone in response to iodine load and is typically managed with antithyroid medications [1]. In contrast, type II AIT, characterized by destructive thyroiditis in an otherwise normal thyroid gland, is generally treated with oral glucocorticoids as first-line therapy. Severe cases, however, may be refractory to medical treatment, even when a combination of beta-blockers, corticosteroids, antithyroid drugs, and supportive measures is used [1]. Lugol's iodine is ineffective in AIT, as the thyroid gland is already saturated with iodine.

In our patient, the presence of a haemorrhagic HCC requiring urgent resection and the high risk of intraoperative thyroid storm warranted expedited control of thyrotoxicosis. These factors justified the use of plasmapheresis as a bridging intervention to facilitate thyroidectomy.

More than 99% of circulating thyroid hormones are protein-bound. Plasmapheresis lowers thyroid hormone levels by removing both the bound and free hormone fractions through redistribution and dilution effects across the intracellular compartment and replacement fluids [9]. Although our patient underwent plasmapheresis a few days after unsuccessful medical therapy, in the setting of thyroid storm, it can be initiated immediately. Fresh frozen plasma (FFP) was selected as the replacement fluid in the final session to minimize coagulopathy risk (presence of clotting factors and immunoglobulins) and to replenish thyroid hormone-binding proteins [2, 10].

The number and frequency of plasmapheresis cycles vary across reports, with most cases utilizing between one and six sessions. The therapeutic aim is to reduce circulating free hormone levels to allow safe general anaesthesia and thyroidectomy [7, 11]. In our case, multidisciplinary consensus determined a relatively safer target of FT4 of <50 pmol/L for surgery. Serial six-hourly thyroid function tests demonstrated a consistent decline in FT3 and FT4 levels following each session, with the reduction attributed to increased binding capacity from proteins introduced via FFP2. A total thyroidectomy was performed within four hours of reaching the target FT4 level (48 pmol/L), without complication.

Plasmapheresis is not without risk and should not be recommended as standard treatment for type II AIT. While effective, plasmapheresis carries risks and should not be considered a standard treatment for all cases of type II AIT. Potential complications include citrate-induced hypocalcaemia, coagulopathies, electrolyte imbalances, and hypotension - all of which require vigilant monitoring by specialized apheresis staff in an appropriate clinical setting [2, 12].

## Conclusions

This case highlights the importance of prompt diagnosis, accurate classification, and timely intervention for AIT, particularly in the context of comorbid conditions requiring urgent surgical intervention. In severe or refractory type II AIT, plasmapheresis can serve as a valuable bridging therapy to definitive thyroidectomy, enabling rapid hormonal control when conventional therapies fail. However, due to its potential risks, this approach is not considered standard therapy for type II AIT and should be discontinued promptly if complications occur. The timing of response, number of exchanges required, and potential complications are variable, reinforcing the need for individualized, multidisciplinary management to optimize patient outcomes.
